# P-464. Implementation of Non-Mydriatic Fundus Imaging for HIV-Associated Meningitis Patients in Uganda

**DOI:** 10.1093/ofid/ofae631.663

**Published:** 2025-01-29

**Authors:** Spencer Yueh, Timothy Mugabi, Kristoffer E Leon, Tu M Tran, David Meya, Caleb Skipper

**Affiliations:** University of Minnesota, Minneapolis, Minnesota; Infectious Diseases Institute, Makerere University, kampala, Kampala, Uganda; University of California, San Francisco, San Francisco, California; University of Minnesota Ophthalmology, Irvine, California; Infectious Diseases Institute, Makerere University, kampala, Kampala, Uganda; University of Minnesota, Minneapolis, Minnesota

## Abstract

**Background:**

Persons with HIV-associated meningitis are at increased risk of visual impairment. It is often unclear whether this is due to the underlying meningitis pathophysiology or whether a second etiology may be present, due to the difficulty in accessing expert exams in resource-limited settings. Our long-term goal is to determine retinal disease prevalence, particularly treatable infections (e.g. CMV retinitis), in those with HIV-associated meningitis. Here we present pilot feasibility data of non-mydriatic fundus imaging in a sick inpatient population in Uganda.

**Figure 1.**
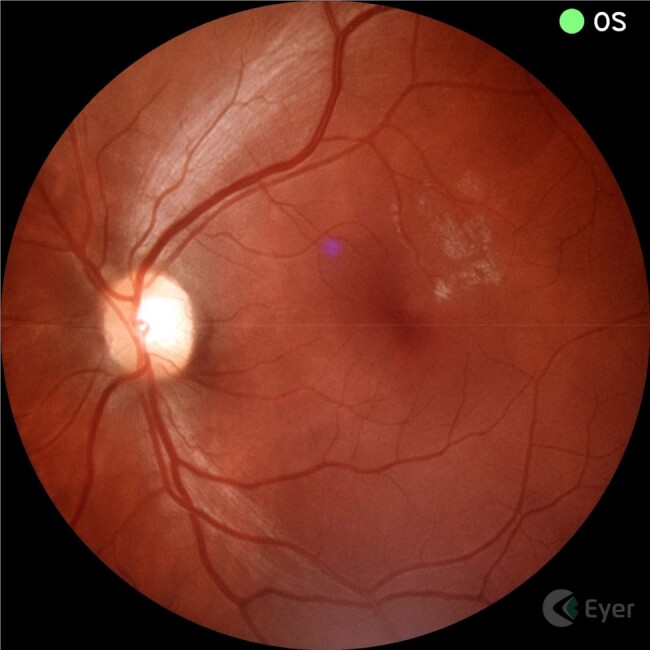
Full FOV image of a healthy retina. Non-mydriatic photo of the left eye of a 31-year-old male admitted for cryptococcal meningitis.

**Methods:**

We designed a prospective cohort pilot study of adult Ugandans with HIV-associated meningitis to determine the feasibility of portable digital fundoscopic photography in detecting retinal pathology. We trained medical officers to use the Phelcom Eyer Fundus Camera, which obtains high quality non-mydriatic images of the fundus. We categorized image quality into three groups: full field-of-view (FOV), partial FOV and interpretable, or uninterpretable photographs with minimal FOV. We determined: 1) the proportion of eyes in which an adequate image is obtained and 2) the prevalence of any retinal pathology in this population.

**Figure 2.**
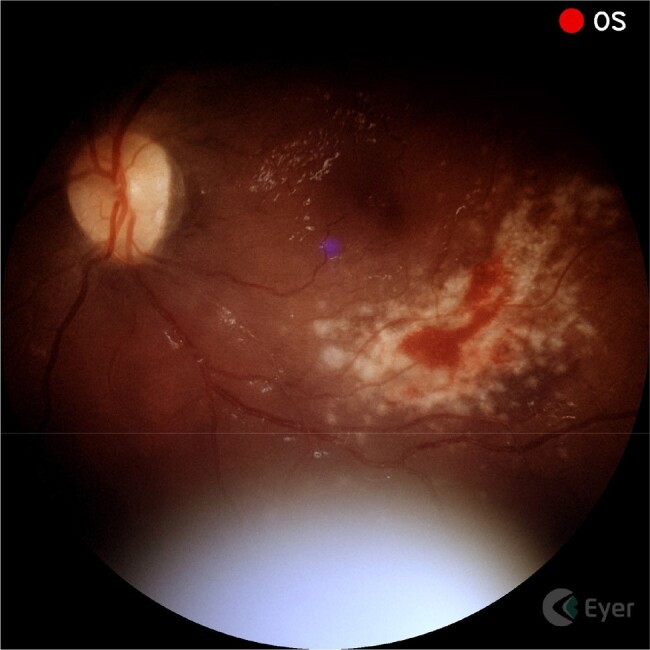
Partial FOV image of CMV retinitis. Non-mydriatic photo of the left eye of a 36-year-old female admitted for cryptococcal meningitis. The fundus has a "cheese and ketchup" appearance, a classic finding in CMV retinitis. (Full case description will be included with the poster)

**Results:**

From 2/15/23 to 5/2/24, we captured retinal images of 245 patients and 475 eyes (15 patients had only one eye imaged), who were hospitalized for HIV-associated meningitis. We obtained a full FOV image (Figure 1) in 62% (296/475) of photographed eyes, while 31% (148/475) of eyes had a partial FOV, and 7% (31/475) of eyes had uninterpretable photographs. Of the 245 patients screened, 75 (31%) showed any retinal pathology. Figure 2 demonstrates a case where non-mydriatic fundus imaging was used to capture and diagnose a reversible case of CMV retinitis.

**Conclusion:**

We demonstrated that the portable Phelcom Eyer camera can successfully generate fundus images to identify retinal pathologies in our patient population. Our team captured interpretable images in 93% of eyes despite the confusion or neurological deficits often present in this sick population. These results demonstrate the necessity of screening for retinal pathology in this population, and that mobile non-mydriatic fundoscopic imaging is a promising solution in resource-limited settings.

**Disclosures:**

**All Authors**: No reported disclosures

